# Disseminated Blastomycosis Misdiagnosed as Community-Acquired Pneumonia

**DOI:** 10.7759/cureus.113040

**Published:** 2026-07-20

**Authors:** Morgan A Allen, Evan Anderson, Irena Medenica

**Affiliations:** 1 Internal Medicine, Rush University Medical Center, Chicago, USA; 2 Neurology, Rush University Medical Center, Chicago, USA

**Keywords:** disseminated blastomycosis, fungal infection, fungal lung disease, invasive fungal infections, pulmonary blastomycosis, respiratory infectious diseases, skin blastomycosis, treatment and diagnosis delay, unique case of blastomycosis

## Abstract

Blastomycosis, caused by *Blastomyces* species, is a fungal infection that can manifest with a wide range of clinical presentations, from asymptomatic cases to severe, multi-organ involvement. Here, we describe a 33-year-old Black woman from an endemic region who experienced progressive, antibiotic-refractory respiratory symptoms and developed a cutaneous lesion, initially misdiagnosed as community-acquired pneumonia. This case highlights the need to include blastomycosis in the differential diagnosis of persistent pneumonia, particularly in at-risk populations, to facilitate timely diagnosis and treatment.

## Introduction

Blastomycosis is a systemic pyogranulomatous fungal infection caused by the thermally dimorphic fungi *Blastomyces dermatitidis* and *B. gilchristii*, both endemic to North America. Although these two species are morphologically similar and produce comparable disease spectra, clinical laboratories do not routinely distinguish between them; consequently, all isolates are typically referred to as *B. dermatitidis* in clinical diagnosis. In the environment, *Blastomyces* exists as a mold, but upon entering human tissue, it transforms into its yeast form, which enables hematogenous dissemination throughout the body [1]. Infection occurs primarily through inhalation of airborne conidia (spores) from the environmental mold phase, as person-to-person transmission has not been recognized. The disease presents along a broad clinical spectrum, ranging from asymptomatic infection to severe, multi-system involvement. Pulmonary disease is the most common manifestation, ranging from mild flu-like illness to severe pneumonia and acute respiratory distress syndrome (ARDS) [2,3]. Dissemination occurs in approximately 25-40% of patients, with cutaneous manifestations occurring in 40-80% of disseminated cases [2,4]. While blastomycosis often affects immunocompromised patients, it can also cause disseminated disease in immunocompetent hosts.

Population-based data from Missouri indicate that Black individuals are disproportionately affected by blastomycosis, with higher incidence, hospitalization, and mortality rates compared to other racial groups. This has led to the Black race being identified as an independent risk factor for endemic blastomycosis, even after controlling for environmental and socioeconomic exposures [5]. The underlying reason for this disparity is unknown; studies suggest a combination of social determinants of health and possible genetic or immunologic factors [5,6]. These disparities highlight the importance of maintaining a high index of suspicion for blastomycosis in minority patients presenting with suspected community-acquired pneumonia, even when immunocompetent. Clinicians in endemic regions should maintain a high index of suspicion for blastomycosis in patients with antibiotic-refractory pulmonary disease, especially within populations that experience a disproportionately high disease burden.

Here, we present the case of an immunocompetent Black woman from an endemic area who developed disseminated blastomycosis with cutaneous manifestations following initial treatment for community-acquired pneumonia. This case highlights the diagnostic challenges associated with blastomycosis in otherwise healthy individuals and emphasizes the importance of considering fungal infection in patients with persistent pneumonia unresponsive to antibiotics, particularly when accompanied by cutaneous findings.

## Case presentation

A 33-year-old Black woman with a history of hypertension presented to the Emergency Department (ED) with progressive shortness of breath, pleuritic chest pain, and intermittent hemoptysis. Her symptoms began five weeks prior to admission with sudden-onset chest pain that awoke her from sleep. The pain resolved spontaneously but recurred several days later, accompanied by a dry cough and flu-like symptoms.

**Figure 1:**
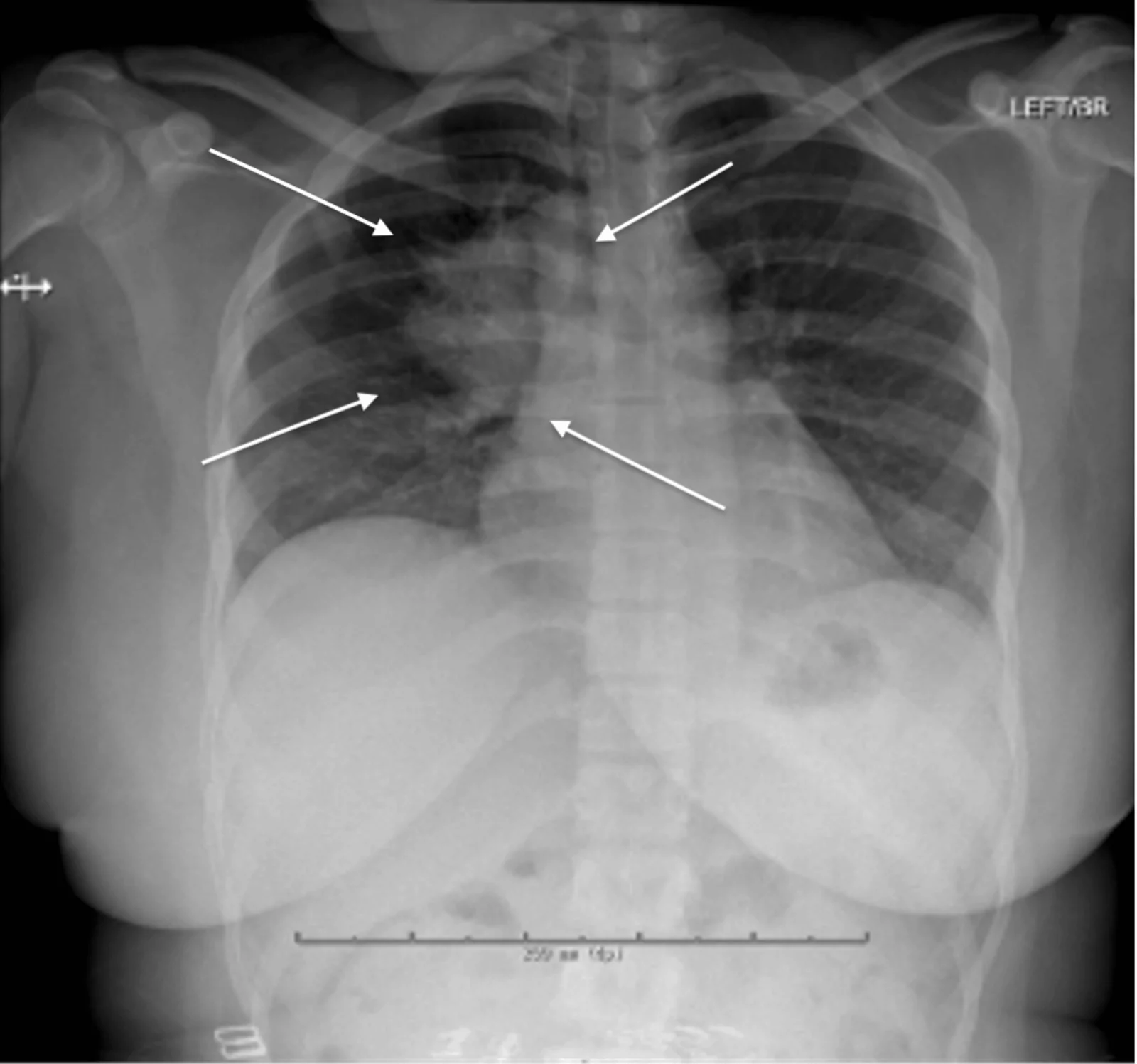
Initial chest X-ray (anteroposterior view). Demonstrating a right hilar opacity concerning focal pulmonary consolidation. Arrows point to the right hilar opacity.

**Figure 2:**
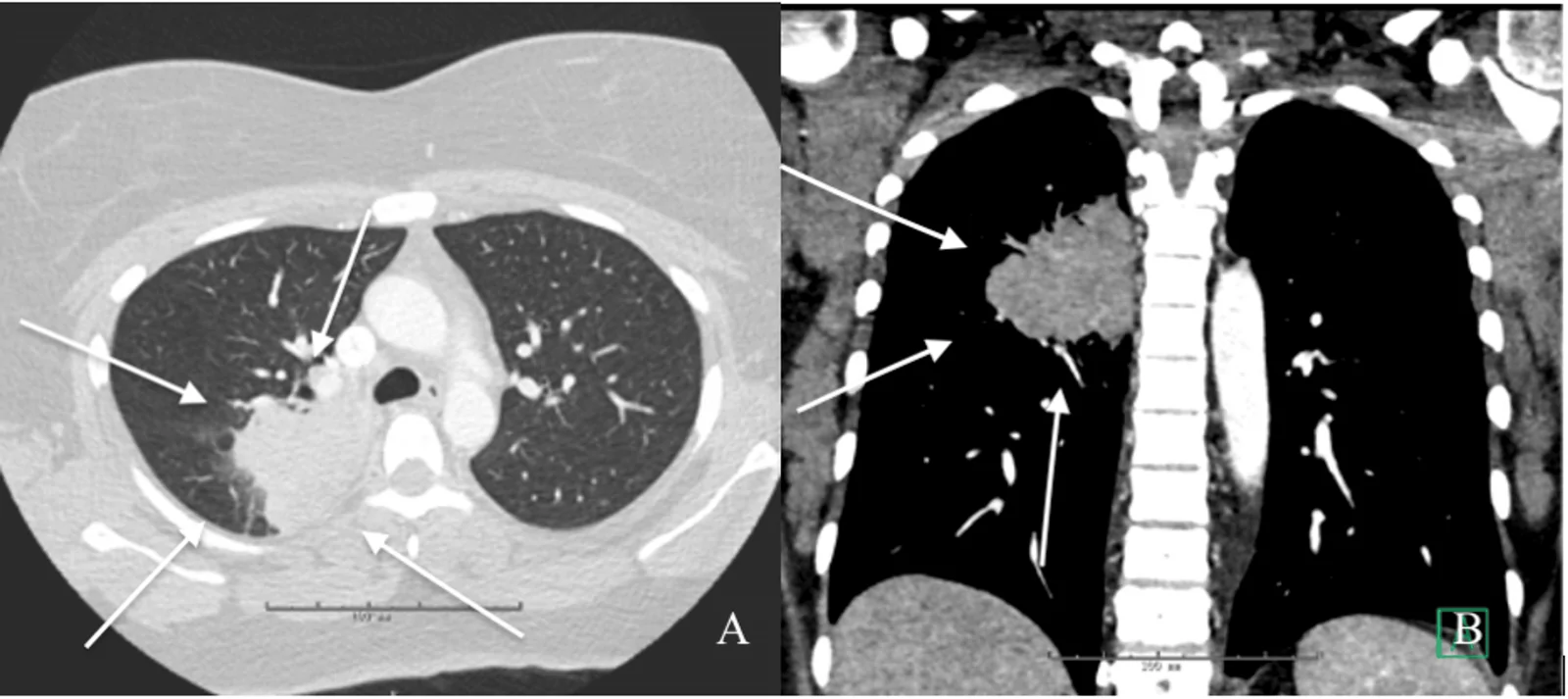
Initial contrast-enhanced CT chest imaging. A: axial view; B: coronal view demonstrating a heterogeneous right upper lobe pleural-based mass-like consolidation abutting the right hilum with adjacent centrilobular nodularity concerning an infectious etiology. Arrows point to a heterogeneous right upper lobe mass abutting the pleura and right hilum.

Approximately one week after symptom onset, she presented to the ED for initial evaluation. At initial presentation, the differential diagnosis included community-acquired bacterial pneumonia, atypical pneumonia, tuberculosis, and pulmonary malignancy. Blood cultures, sputum cultures, and the QuantiFERON-TB Gold assay (QIAGEN, Hilden, Germany) were negative. Chest radiography revealed a right hilar opacity (Figure 1). Subsequent chest computed tomography (CT) showed a heterogeneous right upper lobe mass abutting the pleura and right hilum, with mild adjacent centrilobular nodules suggestive of infection (Figure 2). Based on her age, immunocompetent status, and imaging findings, community-acquired pneumonia was considered the most likely diagnosis, and she was discharged on amoxicillin-clavulanate 875/125 mg orally twice daily for 10 days and azithromycin 250 mg orally once daily for four days. This dual therapy was chosen to cover common respiratory pathogens, including atypicals. She was instructed to follow up to ensure symptom resolution.

Despite adherence to her antibiotic regimen, the patient reported no clinical improvement and experienced worsening dyspnea and cough. She followed up with her primary care physician, whose workup revealed leukocytosis (16.6 K/uL). Inflammatory markers, including C-reactive protein (118 mg/L), sedimentation rate (116 mm/hr), and procalcitonin (0.19 ng/mL), were elevated. Repeat sputum culture with Gram stain showed a few Gram-positive cocci. Her primary care provider prescribed a second course of antibiotics: cefdinir 300 mg orally twice daily for seven days and clarithromycin 500 mg daily for seven days to broaden bacterial coverage. However, over the following week, her condition deteriorated. She developed new symptoms, including fever (101.5°F), night sweats, diaphoresis, orthopnea, anorexia, insomnia, and intermittent hemoptysis.

In the fourth week of her progressive illness, she noticed the development of a painless, enlarging nodule on the dorsum of her right hand. Initially presumed to be a pimple, the lesion rapidly enlarged and became tender to the touch. She also began to experience episodes of cough-induced emesis, prompting her to return to the ED.

**Figure 3:**
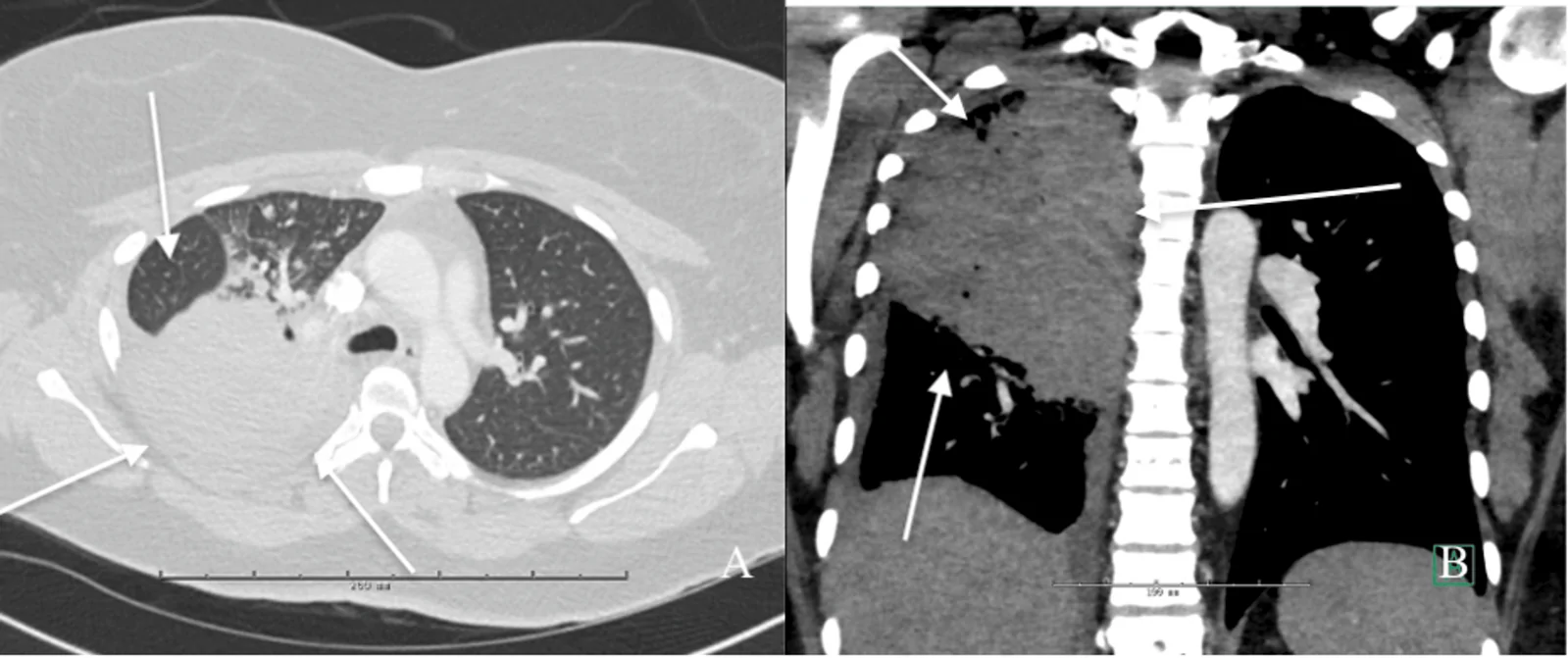
CT angiogram of the chest obtained during repeat presentation. A: axial view; B: coronal view demonstrating interval progression of right-sided pulmonary consolidation involving the upper and lower lobes with internal air bronchograms. Arrows point to the right lung consolidation that now involves both the upper and lower lobes.

On presentation, she was hemodynamically stable but tachycardic (110). Labs were significant for worsening anemia (Hgb 9 g/dL), thrombocytosis (547 K/µL), and leukocytosis (24 K/µL). The mini respiratory pathogen panel in the ED was negative. CT angiography of the chest revealed a significant interval increase in the size of a right lung consolidation, now involving both the upper and lower lobes with internal air bronchograms (Figure 3).

**Figure 4:**
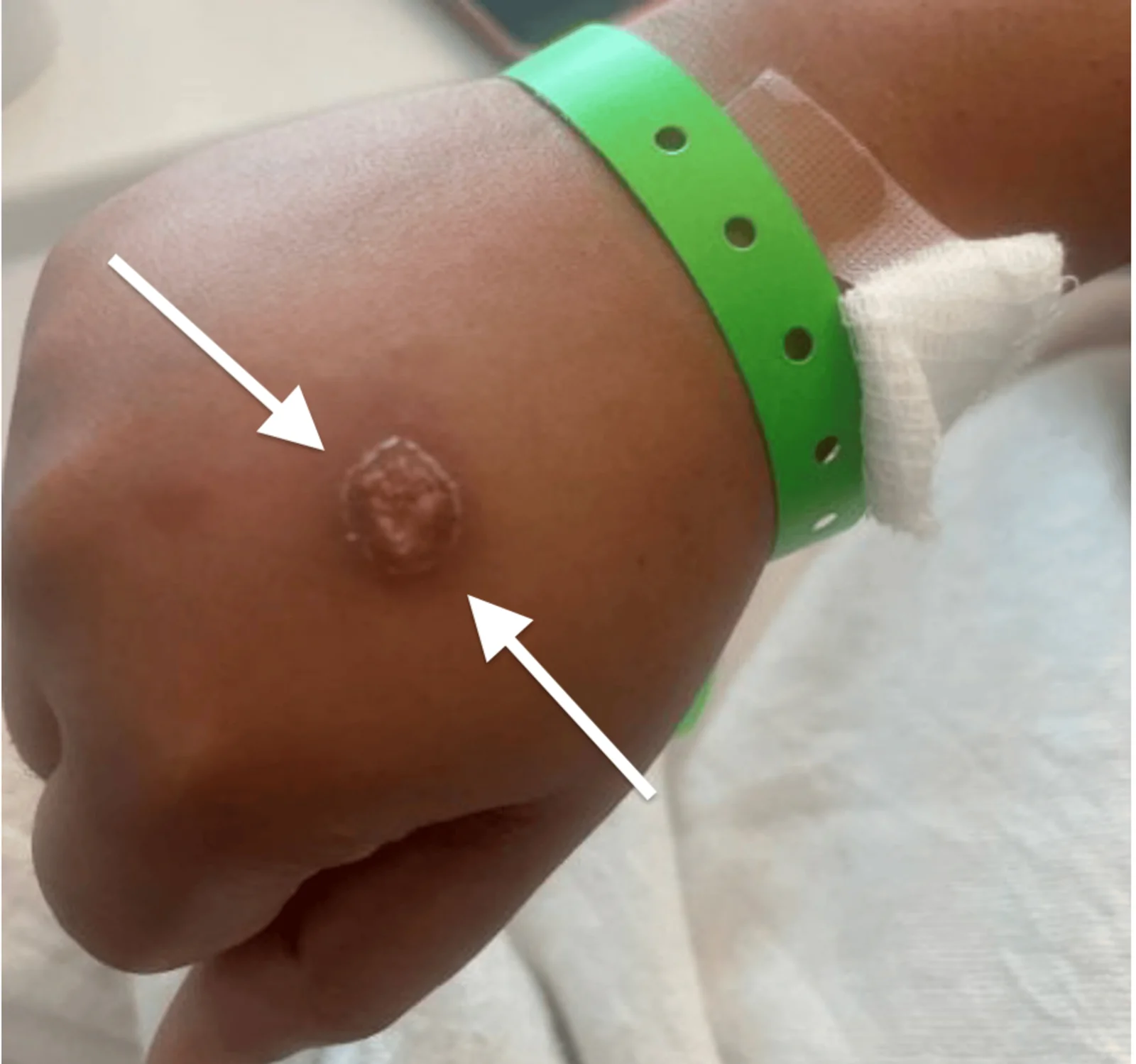
Cutaneous lesion on the dorsum of the right hand prior to biopsy. Demonstrating a tender violaceous nodular lesion associated with disseminated blastomycosis. Arrows point to the right-hand lesion.

Given her clinical deterioration despite broad-spectrum antibiotics and the presence of a concerning skin lesion, a comprehensive infectious disease workup was initiated. This included repeat blood cultures, an expanded respiratory panel, methicillin-resistant *Staphylococcus aureus* (MRSA) nasal swab, acid-fast bacilli culture, sputum culture, and urine *Legionella* antigen testing. All initial tests were negative. Due to the lesion on her right hand (Figure 4), dermatology was consulted, and a punch biopsy with Calcofluor white staining was performed. The biopsy revealed focal surface ulceration and dense dermal suppurative and granulomatous inflammation. Periodic acid-Schiff (PAS) staining demonstrated broad-based budding yeast, confirming disseminated *Blastomyces dermatitidis*. Subsequent cultures confirmed *Blastomyces dermatitidis*, and the Histoplasma urine antigen was positive at an unquantifiable level, likely reflecting cross-reactivity in the setting of disseminated blastomycosis [7].

An infectious disease consultation was obtained, and the patient was started on itraconazole 200 mg orally three times daily for three days, followed by 200 mg twice daily for at least six to 12 months. She was instructed to follow up with the infectious disease specialist in two weeks to assess for resolution of the infection.

At her two-week follow-up, the patient reported marked symptomatic improvement, with persistent but reduced exertional dyspnea. Her cough remained, but was now non-productive. At her six-week follow-up, she reported complete resolution of respiratory symptoms, including dyspnea, cough, and chest pain. The cutaneous lesion on her right hand was also resolving. She will continue itraconazole for at least six months and has a scheduled follow-up to evaluate for complete resolution of disseminated *Blastomyces dermatitidis*.

## Discussion

Traditionally, *Blastomyces* has been considered endemic to the Midwest and regions surrounding the Ohio and Mississippi River valleys; however, its geographic range has been expanding in recent years. The exact reason is still being investigated, but this is thought to be due to environmental changes, increased travel, or potentially increased awareness leading to increased testing and diagnosis [8].

Blastomycosis can pose a diagnostic challenge, particularly in immunocompetent patients, whose symptoms may be less severe and indolent, mimicking other pulmonary or systemic diseases. This often results in misdiagnosis or delayed diagnosis [9,10].

Diagnosis is further complicated by the absence of a single best test with data that supports both high sensitivity and specificity. The Infectious Disease Society of America endorses culture as the gold standard for diagnosis, with methenamine silver or PAS used for extrapulmonary disease [11]. However, culture is slow, often requiring several days to weeks for growth, which delays diagnosis and treatment [1]. Histopathology depends on specimen quality and may not be able to distinguish *Blastomyces* from other fungal elements [12].

Blastomycosis presents along a broad clinical spectrum, ranging from asymptomatic infection to severe, multi-organ involvement. Pulmonary involvement is the most common manifestation, and acute cases often resemble community-acquired pneumonia, with symptoms such as fever, cough, chest pain, and shortness of breath. In contrast, chronic pulmonary disease may mimic tuberculosis or malignancy, with radiographic findings that include mass-like lesions, nodules, or cavitary infiltrates. Hematogenous spread of the yeast form is common and may result in extrapulmonary involvement.

Cutaneous blastomycosis is the most frequent manifestation of disseminated disease, occurring in 40-80% of disseminated cases [2,4,11]. Skin lesions typically present as verrucous, ulcerated, or crusted plaques that can be mistaken for squamous cell carcinoma [2,11]. Other sites of dissemination include the bones (approximately 4%), genitourinary system (around 2%), and central nervous system (about 1%). Unlike other endemic mycoses, cutaneous findings in blastomycosis are often the initial indication of systemic disease [2,12].

Despite its relatively low incidence, blastomycosis is associated with significant morbidity. Severe pulmonary and disseminated forms can lead to prolonged hospitalization and complications [4,13]. Early recognition and initiation of antifungal therapy is essential to reduce complications and improve outcomes [14].

This case demonstrates that blastomycosis may occur in young, immunocompetent individuals without apparent risk factors and can present with features that resemble more common pulmonary and dermatologic disorders. In endemic areas, clinicians should consider blastomycosis in the differential diagnosis of progressive pulmonary disease that fails to respond to antibiotics, particularly when accompanied by cutaneous manifestations. Early recognition and timely diagnostic evaluation are essential to avoid delays in treatment and minimize disease-related morbidity.

This report is limited by its retrospective, single-patient nature and by the absence of long-term radiographic follow-up. Nonetheless, it underscores an important diagnostic challenge and reinforces the need to consider blastomycosis in patients with persistent, antibiotic-refractory pneumonia.

## Conclusions

This case highlights that blastomycosis can occur in young, immunocompetent patients without obvious risk factors, and presentations may mimic more common pulmonary and dermatologic conditions. Clinicians practicing in endemic regions should maintain a broad differential diagnosis, particularly when evaluating progressive, antibiotic-refractory pulmonary disease with associated skin lesions. Heightened suspicion and timely diagnostic evaluation are crucial to preventing delays in care and reducing morbidity, particularly in at-risk populations.
